# Sinonasal and Pulmonary Computed Tomography Images Before and After Triple-Combination Therapy in a Patient with Cystic Fibrosis Without ΔF508 Mutations

**DOI:** 10.3390/diagnostics16111692

**Published:** 2026-05-30

**Authors:** Corrado Tagliati, Giovanna Campagna, Maria Di Sabatino, Giuseppe Lanni, Davide Battista, Pietro Ripani

**Affiliations:** 1 AST Ancona, Ospedale di Comunità Maria Montessori di Chiaravalle, Via Fratelli Rosselli 176, 60033 Chiaravalle, Italy; 2 Dipartimento Materno Infantile, UOSD Centro di Riferimento Regionale Fibrosi Cistica, Ospedale “San Liberatore”, Viale Risorgimento, 64032 Atri, Italy; giovanna.campagna@aslteramo.it (G.C.); maria.disabatino@aslteramo.it (M.D.S.); pietro.ripani@aslteramo.it (P.R.); 3 Dipartimento dei Servizi, UOSD Radiologia Ospedale “San Liberatore”, Viale Risorgimento, 64032 Atri, Italy; giuseppe.lanni@aslteramo.it (G.L.); davide.battista@aslteramo.it (D.B.)

**Keywords:** cystic fibrosis, imaging, lung, sinonasal disease, computed tomography

## Abstract

Here, we present the case of a 25-year-old patient with *G542X* and *G85E* cystic fibrosis mutations who underwent computed tomography examination before and after triple-combination therapy. Clear improvement in sinonasal and lung involvement is visible two years after modulator treatment initiation. To the best of our knowledge, this is the first report about sinonasal improvement demonstrated by computed tomography images in a patient with *G542X/G85E* mutations.

**Figure 1:**
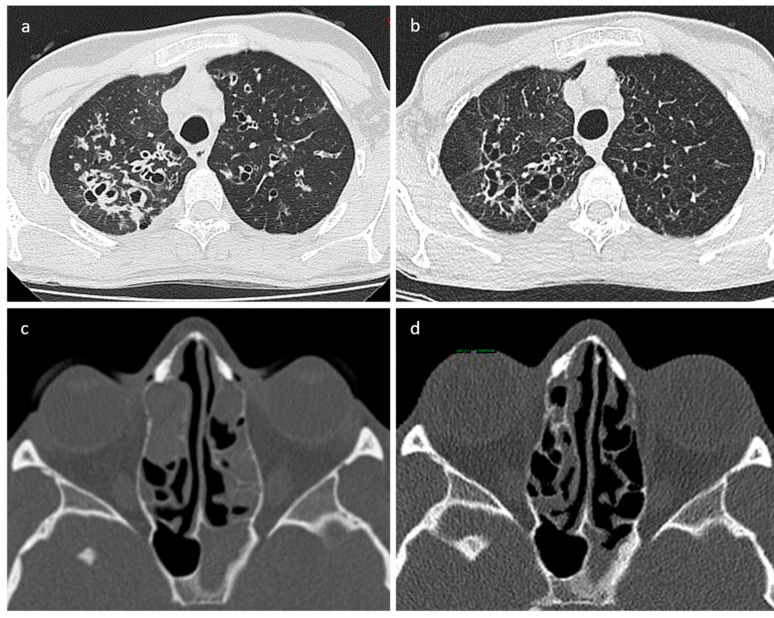
A 25-year-old patient with *G542X* and *G85E* cystic fibrosis mutations performed lung and sinus computed tomography examinations before (**a**,**c**) and after (**b**,**d**) triple-combination therapy. Computed tomography lung and sinus examinations were performed using a 64-slice scanner (Lightspeed VCT 64, GE, Milwaukee, WI, United States) with patients in supine position and head towards the gantry. The lung scan parameters of computed tomography examinations performed in the caudocranial direction in suspended deep inspiration were as follows: scan range from the lung apices to the diaphragm, 64 × 0.625 mm beam collimation, 100 kVp tube voltage, tube current variable between 20 and 150 mAs, noise index 50, spiral pitch factor 1.375, rotation time 0.5 s, matrix 512 × 512, 1.25 mm slice thickness reconstructions and an increment of 1.25 mm, and coronal and sagittal images for multiplanar reconstructions using an adaptive statistical iterative reconstruction (ASIR) protocol with application of 60% ASIR to the raw datasets. The sinus scan parameters were as follows: scan range from the hard palate to above the end of the frontal sinuses, 64 × 0.625 mm beam collimation, 100 kVp tube voltage, 40 mA tube current, 1.25 mm slice thickness reconstructions, and coronal and sagittal images for multiplanar reconstructions, using both bone and soft-tissue reconstruction kernels. Clear improvement in sinonasal and lung involvement is visible two years after modulator treatment initiation. In fact, lung scan after treatment showed mucous plugging and peribronchial thickening reduction, and sinus scan showed paranasal sinuses opacification decrease. For forced expiratory volume in one second, percentage of predicted value was 44 before treatment and 58 two years after its initiation [1,2]. Brody-II score evaluates chest disease involvement assessing mucous plugging, peribronchial thickening, bronchiectasis, parenchyma and hyperinflation, and it was 21 before treatment and 15 two years after its initiation [3]. The not-cystic fibrosis-specific computed tomography modified Lund–Mackay score and the cystic fibrosis-specific Sheikh–Lind computed tomography sinus disease severity scoring system were, respectively, 11 and 9 before treatment, and 8 and 6 two years after its initiation [4,5]. The 22-item SinoNasal Outcome Test questionnaire evaluated sinonasal quality of life impairment, and it was 28 before treatment and 16 two years after its initiation [6,7]. Elexacaftor–tezacaftor–ivacaftor contains two correctors and a potentiator of the cystic fibrosis transmembrane regulator channel [8,9,10]. A few years ago, it was indicated for patients 6 years of age and older who had at least one copy of the *F508del* gene [11]. More recently, it has been authorized for patients aged 2 years old and older who have at least one non-class I mutation [12]. This allows a lot of cystic fibrosis patients to be treated with this highly effective therapy, obtaining clear lung improvements. In fact, elexacaftor–tezacaftor–ivacaftor treatment was associated with lung function improvement, pulmonary exacerbation reduction, improved gas exchange, enhanced nutritional status and lung transplant reduction [13,14,15]. Computed tomography examinations before and after triple-combination therapy initiation showed a significant improvement in structural lung disease [16]. Moreover, triple-combination therapy was associated with sinonasal disease improvement, and a reduction in the need for endoscopic sinus surgery was reported [17,18,19]. Furthermore, previously published articles reported a reduction in sinus disease severity in computed tomography [18,19,20,21,22,23,24]. However, to the best of our knowledge, this is the first report about sinonasal improvement demonstrated by computed tomography images in a patient with G542X/G85E mutations.

## Data Availability

The original contributions presented in this study are included in the article. Further inquiries can be directed to the corresponding author.
